# Principles of good practice for translation of electronic clinical outcome assessments

**DOI:** 10.1186/s41687-025-00859-4

**Published:** 2025-03-04

**Authors:** Huda Shalhoub, M. Turner, A. Bradley-Gilbride, S. Eremenco, H. Muehlan, E. Parks-Vernizzi, B. Arnold, D. Kuliś, C. Anfray, J. E. Chaplin, J. P. Repo

**Affiliations:** 1 Bayer AG, Digital & Commercial Innovation, Pharmaceuticals Pulmonology, Women’s Healthcare & Radiology, Building S157, 313, 13342 Berlin, Germany; 2 GSK, Philadelphia, PA USA; 3 Health Psychology Research Ltd, Egham, Surrey, UK; 4 Critical Path Institute, Tucson, AZ USA; 5 Health and Medical University, HMU, Erfurt, Germany; 6 FACITtrans, Ponte Vedra, FL USA; 7 European Organisation for Research and Treatment of Cancer, Brussels, Belgium; 8 Mapi Research Trust, Lyons, France; 9 Sahlgrenska Academy at Gothenburg University, Gothenberg, Sweden; 10 Tampere University Hospital and University of Tampere, Tampere, Finland

**Keywords:** eCOA, Translation, Linguistic validation, Cultural adaptation, Clinical research, Electronic implementation, Best practices

## Abstract

**Background:**

While many publications have outlined good practice recommendations for translation and electronic implementation of clinical outcome assessments (COAs), they are often treated as independent processes. The scientific literature currently lacks recommended guidelines on the process of concurrent translation, cultural adaptation and electronic implementation of COAs for clinical research. In response to this need, the ISOQOL Translation and Cultural Adaptation Special Interest Group (TCA-SIG) sought to identify actionable steps for addressing the scientific and operational intricacies in this concurrent process.

**Methods:**

Using snowball sampling, semi-structured questions were sent to language service providers (LSPs), electronic clinical outcome assessment (eCOA) providers, and developers/copyright holders. The TCA-SIG workgroup, consisting of 13 members, then led the methodological groundwork for the disseminated surveys and established a cohesive set of recommendations.

**Results:**

The collective feedback that led to the recommendations included a total of 30 experts who responded to the surveys. Most of the respondents worked in companies or represented organizations based in the US and Europe.

**Recommendations:**

The recommendations fall into two main categories: namely, operational and scientific. The operational recommendations consist of active involvement from all stakeholders, the communication of clear expectations from the start, and better clarification of timelines of LSPs involved. Examples of scientific recommendations are electronic language feasibility assessment (ELFA), screenshot proofreading, as well as COA-specific developer and copyright holder guidelines for electronic implementation. COA-specific guidelines and instructions for electronic implementation and evaluation were seen to be needed and key recommendations are discussed in detail in this paper.

**Supplementary Information:**

The online version contains supplementary material available at 10.1186/s41687-025-00859-4.

## Introduction

As the landscape of drug development is becoming increasingly competitive, there is mounting pressure to accelerate research and development [1]. In this dynamic environment, COA selection, validation, translation, cultural adaptation, and psychometric measurement efforts are all significantly impacted. The best practice recommendations outlined in this paper emphasize the rationale for simultaneously translating and migrating instruments electronically. In the past, translation, cultural adaptation, and linguistic validation of COAs were performed independently of electronic implementation, and they were often the rate-limiting step that delayed clinical trial study launch because these processes were conducted sequentially. By adopting a combined approach, the concurrent process ensures that scientific quality and integrity are upheld, meeting the demands of this proactive and fast-paced drug development space.

Clinical research has seen trends in two overarching areas over the last decade; one is the increased involvement of trials that prioritize global perspectives in clinical settings, and the other is the increased use of and reliance on electronic modes to capture patients’, clinicians’, and observer-reported data [2, 3]. These changes have not gone unnoticed by regulatory bodies such as the Food and Drug Administration (FDA) and the European Medicines Agency (EMA). In fact, FDA released multiple guidance documents on patient- reported outcome (PRO) measures and clinical outcome assessments (COAs) over the years, highlighting the importance of developing technical knowledge to inform strategies for the development and use of electronic capture of data from patients [4–5]. Most recently, the FDA Patient-Focused Drug Development (PFDD) [5] draft third guidance also delineates the importance of reviewing translated versions of an instrument that have been migrated to an electronic format. These documents explain that when a measure is being migrated from paper to an electronic format or translated / culturally adapted into another language, additional evidence needs to be provided to confirm the adequacy of the new format or language version of the measure to identify if it has an impact on the COA’s measurement properties. In order to accomplish this faithful migration, the use of pilot testing, in the form of usability testing, and cognitive interviews during eCOA instrument development are suggested. PFDD guidance documents also provide supplementary information about incorporating a translatability assessment (TA) in the instrument development process, further referencing the International Society for Pharmacoeconomics and Outcomes Research (ISPOR) task force reports on principles of good practice for translation and cultural adaptation methods [6].

Other seminal good practice documents have been published by the Critical Path Institute (C-Path) eCOA Consortium, PRO Consortium, ISPOR and the International Society for Quality of Life (ISOQOL) Translation and Cultural Adaptation Special Interest Group (TCA-SIG) are related to either eCOA migration, TAs, or translation best practices in general [7–11]. These publications cover various steps of translation and linguistic validation processes, or the electronic migration of COAs process independently, and remain valid and well-founded when it comes to setting standards for these processes. However, they do not address the increasingly requested concurrent translation and eCOA development practices. Only Eremenco and colleagues [7] briefly mentions electronic implementation of translations, but they take the sequential approach that translations are handled first, then electronic implementation is considered. These considerations do not adequately address the complexity of conducting these processes simultaneously. As a result, while keeping these guidelines in mind and treating them as basis for the concurrent process, the need for this paper crystalized with its specific scope.

Clancy, Crane and Millman (2018) outline in a webinar presentation that there are efficiency gains when integrating eCOA implementation and linguistic validation as there are duplicate processes that often take place, including kickoff, client review, final proofreading, final report and certification [12]. They present an eCOA integration process which includes the following steps: initial project meeting with stakeholders, concept definitions obtained from the COA developer, two forward translations, reconciliation, back translation, resolution, clinician review (if a clinician-reported outcome [ClinRO] measure), screenshot review, cognitive interviews with screenshots, analysis of cognitive interviews, screenshot review resolution, post-localization testing (review of translations on devices and their functionality), and report/certification. Oxford University Innovation’s guidelines on electronic migration [13] highlight that there are additional checks that are needed (e.g., proofreading by a qualified translator) to reduce human error when translations are being deployed electronically. The previously mentioned consensus paper by Eremenco and colleagues’ [7] outlining the PRO Consortium translation process suggested that when translating and electronically implementing a COA, supplementary text translations (e.g., skip alerts, error messages) need to be translated and paid attention to early on. Another vital step they alluded to is screenshot proofreading of the translations between eCOA provider and language service provider (LSP). A consensus panel by Romero et al. [14] also supports the notion of screenshot review by copyright holders for the electronic migration of ClinRO measures when there are minimal modifications from paper. Sweeney and Kelly [15] introduce a process they call a “migration assessment” where eCOA software intricacies can be addressed for different languages. This step is said to essentially reduce the need for re/programming for quality issues, ultimately improving study timelines.

Upon review of the literature, it appears that there is a clear need for scientific and operational guidelines for when a measure is developed electronically and needs translation and/or cross-cultural adaptation, concurrently. An example of a ‘concurrent’ COA development and translation process is when an instrument is planned to be administered electronically, while at the same time it is planned to be translated and culturally adapted in a large number of languages in a clinical study. To bridge this gap, the ISOQOL TCA-SIG established a working group (WG) on “Principles of Good Practice for Translation of Electronic Clinical Outcome Assessments (eCOA)” to: (1) identify any existing translation approaches utilized in eCOA development; (2) review the currently used methodologies and summarize all steps, identifying similarities and differences between approaches, and lastly; (3) define and outline a minimal set of mandatory and optional steps as principles of good practice for eCOA translation. The ISOQOL TCA-SIG eCOA WG collaborated with leading health outcomes contract research organizations, C-Path, pharmaceutical companies, COA researchers, representatives of COA developers and copyright or license holders, and COA LSPs to carry out this endeavor. Of important note, this guidelines paper uses the word translations, but often translations and cultural adaptations are completed together, so we use this languageinterchangeably.

## Methods

### Authorship

The ISOQOL TCA-SIG eCOA WG consisted of 13 experts from a wide variety of industries with significant experience in reviewing, translating, migrating and culturally adapting COAs. Their affiliations include the non-profit sector (Arthritis Research Canada, Critical Path Institute), academia (Health and Medical University Erfurt, University of Tampere, University of Gothenburg), the pharmaceutical industry (GSK, Bayer AG), COA developers, copyright holders and/or their representatives (EORTC, FACIT.org, Health Psychology Research, Mapi Research Trust), and LSPs (FACITtrans, ICON/ Mapi, TransPerfect). Contributions to this work were determined on a voluntary basis from the pool of 187 ISOQOL TCA-SIG members early in its development. Authors on this paper were volunteers who contributed to the work based on authorship rules of the International Committee of Medical Journal Editors (ICMJE). Authors met regularly and collaborated closely to complete this research.

### Creation and distribution of methodology questionnaires

Two web-based questionnaires were developed by the TCA-SIG eCOA WG to collect information regarding the experts’ experience with translations when combined simultaneously with electronic migration and implementation. The target population included (1) LSPs, (2) eCOA providers, and (3) developer / copyright holders. [See Appendices A and B in the online supplementary material for survey questions]. These surveys were administered online via Survey Monkey in English only and managed by ISOQOL administrators. The questionnaires contained a combined total of 47 items in the form of open-ended and closed questions that were developed and tested by the eCOA WG. Each questionnaire required anywhere between 10 and 30 min to complete, depending on the branching logic applicable in each case.

The items in the first survey largely focused on specifics of the translation and cultural adaptation techniques, eCOA intricacies and quality control processes. Topics included the frequency of projects, timelines, copyright and implementation issues, sources of funding, methodological differences between electronic and paper COA projects, process steps required for translation and for cognitive interviews / pilot testing, pre-set requirements, COA developer involvement, best practices, and process considerations specific to paper-to-electronic migration of COAs. The first survey (Appendix A) consisted of a core set of questions that was administered to both LSPs and eCOA providers with branching items by organization type. For example, items for LSPs assumed they carried out the translation work, whereas items for eCOA providers assumed they outsourced the translation work to an LSP. The developer/ copyright holder questionnaire was created with targeted questions related to that stakeholder group’s unique experiences.

A variety of organizations were invited to participate, including LSPs, developers and copyright holders, academics, non-profit organizations, eCOA providers, scientific experts and consultancies, as well as platform providers. The eCOA provider questionnaire was distributed using snowball sampling methods to a total of 13 individuals representing 11 organizations, while the LSP questionnaire was distributed to 8 individuals representing 8 organizations. The developer/ copyright holder questionnaire was also disseminated using snowball sampling, using pre-defined contact lists from the eCOA WG’s organizations. A total of 34 developers and developer / copyright holders were directly approached for survey participation through email. All survey contacts provided consent to receive the survey and authors followed. General Data Protection Regulations to disseminate the survey (e.g. voluntary participation, informed decision, easy withdrawal) (GDPR Art. 13 [16]).

The LSP and eCOA provider questionnaires were disseminated and completed online between September 2019 and October 2019, while the developer / copyright holder questionnaire was disseminated and completed between October and November 2021. In addition to discussions with the TCA-SIG members, the key findings from the questionnaires were discussed with the Critical Path Institute’s eCOA Consortium to ensure representation of all stakeholders involved in COA-specific drug development and a range of perspectives in the findings and recommendations.

### Overview of the expert sample completing the questionnaires

A total of 30 globally representative experts provided their input into the questions; 18 eCOA and LSP providers participated in the first survey, and 12 developers / copyright holders participated in the second survey.

#### eCOA and LSP provider survey overview

After data cleanup, the analysis sample included a total of 18 experts who completed the LSP and eCOA survey (duplicates and incomplete responses removed, and questions were completed voluntarily). Almost half of the sample (39%; *n* = 7/18) included experts representing eCOA providers (Clinical Ink, Kayentis, Medidata, My Clinical Outcomes, Science37, Signant Health, and YPrime); a third (33%; 6/18) worked for LSPs (FACITtrans, ICON Language Services, SDL, Mapi Trust, RWS Life Sciences, and TransPerfect), and the remainder (28%; 5/18) represented three COA consulting companies (Evidera, Oxford University Innovation, and Vitaccess) and two non-profit organizations (Critical Path Institute and Mapi Research Trust). The majority (72%; *n* = 13/18) of the experts’ day-to-day eCOA/translation/consulting work was reported to be undertaken in both US and non-US regions. The company headquarters were based in Europe, North America, and Southeast Asia.

#### Developer / copyright holder survey overview

Ten developers / copyright holders and two scientific experts involved in the process fully completed the copyright holder survey. The majority (83%; 10/12) of the participating experts worked for organizations that owned the license for at least four COAs. The majority (75%; 9/12) of experts reported up to four weeks as the time needed for licensing a COA, from start to finish. The others were either not sure or reported needing 13 weeks or more. The participating experts represented the following companies/organizations: Canadian Memorial Chiropractic College, Critical Path Institute’s Patient-Reported Outcome (PRO) Consortium, FACIT.org, GSK, Mapi Research Trust (MRT), Northwestern University / HealthMeasures / PROMIS, QualityMetric Incorporated, LLC, and Universidad Autónoma de Madrid. Company headquarters are located in the following countries: Canada, France, Spain, the United Kingdom, and the United States. All 12 participants have had direct involvement in the electronic migration of COAs. Almost all (92%; 11/12) had worked with the COA migration in the source language, followed by existing (67%; 8/12) or new translations (67%; 8/12).

## Results

### eCOA/ LSP survey results

#### Existing approaches to translation / eCOA implementation

When experts were asked who is involved in the electronic migration and translation of the same COA, the following roles were reported: project manager/coordinator (94%; 17/18), eCOA expert (89%; 16/18), linguist (72%; 13/18), scientific expert (56%; 10/18), cultural and clinical expert (44%; 8/18), and research scientist (33%; 6/18). Slightly more than half (56%; *n* = 10/18) reported involving additional staff as well (not listed in the survey /not asked to specify). Almost all experts (94%; 17/18) reported having implemented eCOA or translation quality checks and/or processes implemented throughout a project lifecycle. The most mentioned (by a third of the sample) was proofreading and checking the electronic translation content against the source translation (see Table 1). Other quality check processes mentioned by at least one expert were using a mapping document of the English source to compare differences between the paper and electronic versions, device testing, user acceptance testing (UAT) and working closely with the developer / copyright holder to follow their requirements in the translation and screenshot proofreading process, first translating the content from the paper format then migrating to the eCOA format.

In addition to quality check processes that providers put in place, 67% (12/18) of the experts reported conducting feasibility assessments to migrate the COA electronically (e.g., select versus circle response in instructions). Two experts (11%) further reported conducting a translatability assessment, with eCOA providers mentioning that they often ask their LSP to conduct this work on their behalf.

**Table 1:** Existing approaches to quality control and/or process checks

Quality control checks and/or processes undertaken when concurrently migrating to eCOA and translating*	Number of times mentioned (*n*)
Migration screenshots/reports are proofread, and quality checked against translation sources	6
eCOA device functionality testing is accomplished; user acceptance testing (UAT)	2
Work with developer / copyright holder to ensure same standards are used, with checking of screenshots with developer/ copyright holder	2
Mapping document created to compare differences between source (e.g., English) and other language versions	1
Translate the document first, then perform eCOA migration	1

*Not mutually exclusive. (*N* = 18)

The majority (72%; 13/18) of experts reported sometimes or often receiving instructions from the developers or copyright holders, whereas 22% (4/18) reported rarely receiving any instructions. All (100%; *n* = 18) experts agreed that it would be helpful to receive specific instructions on electronic migration from developers of the COA in question. A third of the experts (35%; 6/18) said that the developers were always involved, another third (29%) said often involved and lastly, a third (29%; 5/18) reported developers were sometimes involved. When migrating a standardized COA to electronic format, three (18%; 3/18) stated that they always involve the developer (24% often, 4/18; 53% sometimes, 10/18). Similarly, only one (6%; 1/18) participant reported always involving the developer during and after electronic migration (35% often, 6/18; 41% sometimes, 7/18). When the developer is involved, experts reported a range of one to three rounds of review and an average of two rounds. When rounds of review take place with an eCOA provider, responses ranged from one to four rounds of review, with an average of two rounds. Moreover, almost all of the experts (94%; 17/18) reported that they at least often or sometimes receive a formattable version (Word or another editable format) of the COA, and all agreed that this is at least somewhat helpful.

The steps undertaken when working on a translation and conducting a migration at the same time for a COA are summarized in Table 2 below. It shows that a feasibility assessment to migrate to electronic COA formats (67%; 12/18) is the most often utilized undertaking, followed by cognitive interviews with the target population (61%; 11/18) and in-country reviews (56%; 10/18). Table 2 also displays the reporting of how often an organization also translates the electronic navigational text prompts (e.g., ‘forward’ or ‘back’ buttons on a screen) showing that a single forward translation (72%; 13/18) is the most common activity. The other translation, review or validation activities related to the electronic navigational text prompts do not seem to happen as often (all less than 50%). The one difference in reporting in the sample is that 2 of the 7 LSPs (12% of total sample) reported conducting a translatability assessment (TAs) on navigational text prompts, while no one indicating conducting TA in the eCOA provider group (*n* = 11).

**Table 2:** Translation and eCOA implementation process

Steps undertaken by experts	Translate and migrate a COA*		Translate electronic navigational text prompts	
	*N*	%	*N*	%
Feasibility assessment to migrate to electronic COA	12	67%	4	22%
Cognitive interviews (CI) with target population	11	61%	4	22%
In-country review	10	56%	6	33%
Developer review	9	50%	6	33%
Back translation review	9	50%	4	22%
Dual forward translation	9	50%	1	6%
Harmonization	8	44%	2	11%
Reconciliation of forward translation	8	44%	1	6%
Single backward translation	8	44%	5	28%
Reconciliation of back translation	6	33%	1	6%
Clinical review	6	33%	3	17%
Translatability assessment (TA)	5	28%	2	11%^T^
Single forward translation	4	22%	13	72%
Dual back translation	3	17%	0	0%
*Other step not listed* *(e.g., quality control process)*	5	28%	7	39%

*Not mutually exclusive. (*N* = 18)

^T^ Only reported by Language Service Providers (LSPs)

Participants chose only the relevant response options; the denominator is not constant

#### Expert opinions on eCOA/ translation research processes and timings

Almost half (42%; *n* = 7) of those who answered this question (*n* = 17) reported that carrying out eCOA migration and translation takes longer than translations of paper-based COAs, while a quarter (24%; 4/17) reported no difference; the remaining third (29%; 5/17) were unsure. A few reasons were noted for the longer timeline including programming time, screenshot proofing, as well as additional proofreading steps. There was a wide variety in timing of implementation by providers across all companies, ranging from an additional 2 weeks to 8 to 10 weeks, and even several months. The top factors reported to result in longer timelines were approval time from clients / other stakeholders (65%; 11/17), programming of the COA (59%; 10/17), the vendor (47%; 8/17), and quality controls (41%; 7/17).

#### Research

To review the currently used methodologies, experts were asked to report the steps their organization undertakes when working on translation and migration, as well as the methods used to evaluate conceptual equivalence. As shown in Table 3, cognitive interviews were the most common form of evaluation (76%; 13/17), followed by usability testing (59%; 10/17) and in-person interviews (59%; 10/17). More than half (59%; 10/17) reported using paper copy screenshots to present the COA content. Of those who reported conducting cognitive interviews for the purpose of linguistic validation or to evaluate comparability between paper and electronic formats, all shared that a typical sample size to conduct this research is 5 to 10 participants. Translation providers more often reported a sample size of 5, while the eCOA providers and scientific experts reported up to 10 more often. For those expert providers who re-test, 50% use a combination of new and existing participants, 30% use new participants, and 20% use the same participants. Moreover, in the majority of cases (88%; 15/17), the experts confirmed that they closely match the specific trial population of interest to the cognitive interview study.

**Table 3:** Research undertaken with translations and eCOAs

COA validation research methodology most used	*N*	%
Cognitive interviews*	13	76%
Usability testing (e.g., tablet, handheld, web)	10	59%
In-person interviews	10	59%
Concept elicitation	3	18%
Telephone interviews	2	12%
Focus groups	1	6%
*Other: Translatability assessment, provider emulator screen, eBooklets or other instructions/ guidelines created*	3	18%

*Responses are not mutually exclusive (*N* = 17)

### Developer / copyright holder survey results

On a 5-point scale ranging from very important to not at all important, the majority (83%; 10/12) of the experts responding to this question indicated it is very important for them to be involved in the electronic migration process. In contrast, although all participating experts stated they are involved in the process, only a quarter (25%; 3/12) reported they play an active role all the time, whereas 25% (3/12) and 50% (6/12) are involved for most of the time and sometimes, respectively. Almost all (92%; 11/12) reported they are involved in electronic implementation (or migratability) assessments of necessary changes to layout, screenshot review and final review after implementing all changes. A large majority (83%; 10/12) are involved in the migratability assessment of necessary changes for electronic migration to content (e.g., changes in wording of instructions, navigational text prompts) and 75% (9/12) play a role in the initial discussion and preparation before the migration process begins. Fewer experts indicated being involved in proofreading migrated translations (67%; 8/12), in the review of usability testing / expert review results (50%; 6/12) and only a third in the review of cognitive interview results.

Most (83%; 10/12) have a set of requirements or recommendations they always share with the requestor (e.g., sponsor, CRO, researcher). In addition, the participating experts indicated that they also share their recommendations with licensees (78%; 7/9), eCOA providers (78%; 7/9), and LSPs as well (56%; 5/9). These requirements include instructions on how to adjust wording for electronic implementation in 86% (6/7) of cases and instructions on adjustments for device screen size or font size in 70% (7/10) of cases.

Although 75% (9/12) of experts reported that they receive screenshots for review at least sometimes, only a quarter (25%; 3/12) receive them all the time. A large proportion of developers / copyright holders (67%, 8/12) observe deviations from the requirements they have for electronic migration (33%; 8/12 sometimes, 16%; 2/12 most of the time, and 16%; 2/12 all the time) and in 67% (8/12) of cases, these discrepancies seem to have resulted from limitations of eCOA platforms to some degree (8%; 1/12 all the time, 8%; 1/12 most of the time, and 50%; 6/12sometimes).

The discrepancies most often observed related to migration for delivery via an electronic platform including unapproved modifications to item wording or response options (78%; 7/9), presentation of copyright information (78%; 7/9), presentation of instructions / recall period (67%; 6/9), and approved modifications in wording of COA instructions (e.g., summarizing to fit screen size (44%; 4/9). In terms of formatting, common problems arise in the areas of the layout of response options (90%; 9/10), text formatting (60%; 6/10), number of items presented at any one time per screen (60%; 6/10) and scrolling, zooming in and out to be able to see all response options on one screen (50%; 5/10). Regarding questionnaire flow, complications most commonly arise for skipping items, if allowed (72%; 5/7) followed by answering missed items (43%; 3/7). A great majority of COA developers provide acceptable alternatives (88%; 7/8) and 25% (2/8) would refuse to allow COA migration if acceptable alternatives are not / cannot be implemented.

Developers / copyright holders reported finding it important to be involved in the final review after implementing changes (64%; 7/11), the initial discussion/preparation phase before the migration process begins, including the preparation of the text specific for electronic migration, which needs to be translated (55%; 6/11), TA of necessary changes for linguistic implementation of content, e.g., changes in wording of instructions, navigational text prompts, their ease of translation (55%; 6/11), screenshot review (55%; 6/11), review of cognitive interview results (45%; 5/11), electronic implementation assessment and proofreading of migrated translations (27%; 3/11) and review of usability testing results (27%; 3/11). When migrating and translating a licensed COA, 82% (9/11) of experts would find a consensus guideline of best practice recommendations useful.

## Discussion

Based on the TCA-SIG eCOA WG survey findings and expert discussions, a set of principles of good practice when translating and implementing electronic formats of COAs at the same time are laid out in this section. The recommendations are divided into operational and scientific action points. The eCOA WG aimed at a realistic, but rigorous process, involving a multitude of stakeholders. It is important to note that similar guidelines would apply in case the COA is developed electronically at the outset; essentially, when not requiring electronic migration from paper formats.

### Recommendations

Table 4 summarizes the operational setup recommendations, starting with actively involving all key stakeholders required for the work. The stakeholders would include translation and eCOA experts, as well as project management and scientific or academic experts, developers / copyright holders and process initiators (e.g., pharmaceutical companies or academics). The patient voice throughout the research is beneficial, so it is also recommended to consider adding patient representation when time and resources permit. Incorporating not only a patient perspective, but also a global perspective can add value as well. This includes considering key research teams, patients, and other stakeholders from different regions around the world, for example. This could provide a more culturally sensitive and well-rounded approach to COA implementations.

**Table 4:** Recommended operational steps for eCOA translation research (when occurring concurrently)

Step	Detail	Priority
Active Involvement of Key Stakeholders	Involve all stakeholders in the eCOA and translation process from start to finish. This includes experienced project managers (eCOA and translation PMs, and/or consulting PMs), the developer / copyright holder, eCOA provider, LSP, sponsor, and scientific expert where involved. Patient representatives can further add value. Having a kick-off and regular meetings are essential.	High
Communication of Clear Expectations	Clear channels of communication and expectations by each party should be established from the outset to facilitate a more efficient and satisfactory outcome. Each stakeholder presents the plan and timeline during kick-off call, for instance.	High
Attention to Timelines	Expected implementation timelines require detailed planning upfront as there are many steps involved (e.g., licensing, eCOA programming, migration, proofreading, language harmonization). Adequate time for the developer / copyright holder and sponsor review should be discussed across stakeholders and factored into timelines accordingly. This includes time necessary for the eCOA provider to implement suggested changes when applicable. Multiple rounds of review should be expected and accounted for.	Depending on circumstance(s)

Abbreviations: eCOA: Electronic clinical outcome assessment, PM: Project manager; LSP: Language service provider

Secondly, communication of clear expectations, while paying close attention to timelines and dependencies, should also be addressed at the initiation of eCOA translation work (e.g., during the project kick-off meeting). In so doing, negotiation of expectations occurs early in the process without compromising the scientific integrity of the research. Although these steps are logical and commonsense to occur for any eCOA implementation, they are especially crucial with concurrent translation and eCOA implementation projects due to the additional steps necessary for the process. Timelines for eCOA providers and LSPs, as well as developer / copyright holder approval timelines vary greatly, making these steps vital for the success of a project. When mapping the timelines for deliverables, it may be beneficial to allow time for, at minimum, two rounds of review. The timelines for both electronic implementation and translation requirements are to be mapped out simultaneously and agreed upon with the core stakeholder team.

The operational steps should be implemented alongside the scientific steps found in Table 5, following involvement of key stakeholders. After multiple eCOA WG discussions and feedback from the survey, the consensus was that starting with an electronic language feasibility assessment (ELFA) of the migrated COA or the electronic implementation would be advantageous. The ELFA would review the master eCOA and the remaining translations (ideally as screenshots or electronic on device) carefully to detect any issues that may arise. The ELFA would also include a review of the electronic navigational text prompts (e.g., back and next buttons). The ELFA would be reliant on translated screenshots or electronic formats of the COA on the device. The ELFA would ultimately include LSPs and eCOA providers, and a scientific consultant if and where possible. Further proofreading of the screenshots by the sponsor (including in-country language reviews) can add another layer of scientific integrity. The eCOA provider can initiate the ELFA process by sending the original source (e.g., English) language screens to the sponsor for review and ultimately, approval by the developer / copyright holder. The additional language screens/screenshots would be sent and reviewed by the LSP where any deviations in equivalence of meaning or content would be highlighted. LSPs would, moreover, pay close attention to COA page titles, screen instructions, bolding, coloring and underlining changes per language.

**Table 5:** Recommended scientific steps for eCOA translation research (when occurring concurrently)

Step	Detail	Priority
Electronic Language Feasibility Assessment (ELFA)	• An electronic implementation feasibility assessment (i.e., migration feasibility assessment) is recommended for the target languages to identify necessary intricacies in content and formatting changes, including navigational text prompts per language. Discuss this ELFA early and ensure a translation and review of screenshots is included. Involve LSPs, eCOA providers, and/or scientific consultants who have expertise in this area.	High
Proofreading	• eCOA provider sends original source (e.g., English) language COA screens/screenshot to, at minimum, the sponsor for review and approval by the developer / copyright holder. • The additional language screens/screenshots should be sent to their LSP. Proofreading by the LSP and sponsor (where applicable, in-country reviews) of all migrated COAs in screenshot mode can take place for all language versions. A third-party provider (e.g., health outcomes scientist or expert) may be used. All parties should follow the developer’s / copyright holder’s guidelines regarding changes. If desired, the developer / copyright representative can be involved in the screenshot review. • LSP is expected to facilitate proofreading of the draft screenshot translation(s) against the original source COA. • Overall visual harmony and review of all languages on screens (electronic implementation assessment and translations on screen) should be assessed by LSP to review any deviations prior to implementation. • It is important that eCOA providers supply draft screenshots to LSPs to identify deviations from source prior to implementation. • LSPs should pay particular attention to COA titles, screen instructions (navigational text prompts), bolding, coloring, and underlining. • If cognitive interviews are planned for each language source, ensure eCOA versions are tested at least as screenshots or on the device itself, if possible. In-country reviews or patient representative reviews per language can be used in lieu of cognitive interviews for electronic migration.	Medium
eCOA Provider Guidelines	• Provide examples of COAs previously approved and integrated into the eCOA provider’s platform to the developer / copyright holder to conceptualize how items may appear in the prospective eCOA platform. May consider drafting the COA in the eCOA system and providing that example as well. It is important to abide by copyright laws when creating examples by obtaining permission from the developer / copyright holder. • Share intricacies about language implementation in eCOA for each language planned. (For example, German may lead to longer screen text vs. English). • Follow developer / copyright holder guidelines for screenshot translations. • Ensure that developers / copyright holders review and approve all migration changes made to the COA. • Share details on mode (i.e., device type), screen size, and screen resolution needs.	High
Developer/ copyright Holder Guidelines	• Require final screenshot review of original source document implementations (additional languages optional). • Provide general written guidelines of expectations related to eCOA screens and translation implementation as needed. This can include screen layout rules for each mode, or how to split or replicate instructions for instance. Include, at minimum, original source version guidelines of the migration instructions. • Consider creating a ready-to-use original electronic format in an editable file (e.g., Excel, Word, Rich Text Format) to facilitate appropriate development or adjustment of translations (i.e., paper COA vs. eCOA) for harmonization purposes. It is important to be careful with version numbers as versions often indicate a revised COA.	Medium

Abbreviations: eCOA: Electronic clinical outcome assessment; ELFA: Electronic language feasibility assessment; LSP: Language service provider

The results divulge that not all translation and cultural adaptation of COAs include cognitive interview research with patients, although it is recommended here. Although displaying the screens in their original format is the ideal scenario (e.g., on smartphone or tablet), it is not always feasible or realistic, therefore, reviewing paper screenshots that are identical to the intended device screen can be used instead. To what extent developers of the original COAs are involved in the translation process appears to vary by organization, but they are reported to be often involved. A fine line between electronic screen practicality and conceptual equivalence of translations would be recommended and should be discussed with all parties. To ensure this is achieved appropriately, an option of conducting cognitive interviews with patients in the native language for a selection of languages (*n* = 5) would be useful. In-country reviewers or patient representatives can be used as acceptable review strategies in lieu of cognitive interviews. Cognitive interviews to assess the integrity of the COA’s instructions, items, and response options in the original language would be assumed to have been done separately and are not being addressed in this paper. UAT is also assumed to have been done separately and is not discussed. Concept elicitation, usability testing and UAT are essentially expected to have been achieved in the source language andUAT is discussed at length in Gordon et al. best practice recommendation paper [17].

It is clear from the results of the survey and expert discussions that there is also a need for eCOA instructions, guidelines, and more specific follow-up during the eCOA development process. Each eCOA provider platform is unique and handles migration or implementation differently, thus possibly leading to inconsistent electronic implementations. For example, in some platforms text cannot be underlined, but it can be bolded; other platforms cannot add more words or need to minimize the font size for specific languages to fit on one screen. Prior to the kick-off meeting, eCOA providers are encouraged to detail the specific intricacies and limitations of their system, including screen size details to guide the overall implementation process as accurately as possible. One simple way to do this could be to share de-identified examples from previous eCOA implementations on their platform, alongside device type details (e.g., screen size).

eCOA providers are also expected to share with developers / copyright holders any changes they make to the original COA to ensure that conceptual equivalence is still maintained. Any changes must be discussed and approved by developers and copyright holders. In addition, it must be highlighted that some changes can only be accepted by conducting a comparability study [18] (e.g., cognitive interviews, usability testing, and/or psychometric testing, depending on the level of change made). There are two further aspects to consider: the scientific impact that should be addressed with the developer, and the copyright impact.

Many developers and copyright holders had concerns that there was little or no communication with eCOA providers after providing approval for licensing and using the COA. This lack of communication was reported to have resulted in alterations of COAs during the implementation process by the eCOA provider without prior knowledge or approval from the copyright holder. Such unapproved changes invite risk that the COA will no longer perform comparably to the COA source document and could negatively affect the validity and reliability of the COA.

The TCA-SIG eCOA WG additionally heard on multiple occasions that eCOA platforms are not always flexible enough to allow the exact implementation of the developer / copyright holder instructions. In such situations, and as a rule, involving developers and copyright holders is essential in the electronic implementation process and source development. At the same time, it was reported that there was a lack of developer / copyright holder instructions that involve both electronic migration and translation. In light of this finding, developers / copyright holders are encouraged to provide general instructions on the best implementation strategies for their COA. This should include, at minimum, the original source language guideline instructions for electronic migration as a ready-to-use original file document. Providing editable files for eCOAs in Excel, Word, or.rtf (Rich Text Format) is preferred as they reduce timelines and potential errors when rekeying text. Developers and copyright holders may want also to require final screenshots (at least for tracking/informational purposes if/when the developers are not involved in the process). Paying close attention to version numbers will be an advantage as they often indicate a revised COA over time.

While the provided general recommendations and processes apply to all COA types, including patient-, observer-, clinician-reported, and performance outcome (PerfO) measures, there are specificities for clinician-reported and performance outcome-based measures, for instance, that are not addressed in this paper. PerfO and ClinRO measures are not always easily electronically and equivalently migrated, and direct research with patients or observers may not be appropriate or even required. Specific attention should be paid during the ELFA process on such details, and these would be discussed early in the project implementation. In such cases, scientific expert advice will be highly recommended. Additionally, when time and resources permit, pilot testing on the eCOA device with patients or observers in different languages can be advantageous but is not mandatory.

## Conclusions

The TCA-SIG recommendations in this paper address the implementation strategies that go beyond traditional COA validation research; they focus on what is becoming more common practice, developing, or migrating a COA electronically and translating and culturally adapting the measure at the same time. Since the drug development landscape is becoming more competitive, research and development are now expected to accelerate faster than ever [1]. These trends have a direct impact on COA selection, validation, translation, cultural adaptation, and psychometric measurement efforts. The outcomes of this research provide best practice recommendations for the combined process of developing or migrating and translating an instrument at the same time to ensure that scientific quality and integrity continue to be met in the new proactive space of drug development. Lastly, it is important to note that the recommendations in this paper are meant to complement existing sources that provide detailed explanations on how to conduct research on ‘fit for purpose’ measures and beyond [6–11].

## Electronic supplementary material

Below is the link to the electronic supplementary material.

Supplementary Material 1

## Data Availability

By request to the corresponding author.

## Abbreviations

CE: Concept elicitation
CI: Cognitive interview
ClinRO: Clinician-reported outcome
COA: Clinical outcome assessment
eCOA: Electronic clinical outcome assessment
ELFA: Electronic language feasibility assessment
EMA: European Medicines Agency
FDA: Food and Drug Administration
ISOQOL: International Society for Quality-of-Life Research
LSP: Language service provider
PerfO: Performance outcome
PRO: Patient-reported outcome
TA: Translatability assessment
TCA-SIG: Translation and Cultural Adaptation Special Interest Group
UAT: User acceptance testing
WG: Working group

## References

[1] Bak A, Burlage R, Greene N, et al (2024) Accelerating drug product development and approval: early development and evaluation. Pharm Res 41:1–6. 10.1007/s11095-023-03566-1 37552384 10.1007/s11095-023-03566-1

[2] Jeong S, Sohn M, Kim JH, Ko M, Seo HW, Song YK, Choi B, Han N, Na HS, Lee JG, Kim IW, Oh JM, Lee E (20221) Current globalization of drug interventional clinical trials: characteristics and associated factors, 2011–2013. Trials 18: 288. 10.1186/s13063-017-2025- 10.1186/s13063-017-2025-1 PMC5480138 28637515

[3] Coons SJ, Eremenco S, Lundy JJ, O’Donohoe P, O’Gorman H, Malizia W (2015) Capturing Patient-Reported outcome (PRO) data electronically: the past, present, and promise of ePRO measurement in clinical trials. Patient 8:301–309 25300613 10.1007/s40271-014-0090-z PMC4529477

[4] US Department of Health and Human Services, Food and Drug Administration, Center for Drug Evaluation and Research, Center for Biologics Evaluation and Research, and Center for Devices and Radiological Health (2022) Guidance for industry: Patient-reported outcomes measures: Use in medical product development to support labeling claims. http://www.fda.gov/downloads/Drugs/GuidanceComplianceRegulatoryInformation/Guidances/UCM193282.pdf. Accessed 22 Oct 2023

[5] US Department of Health and Human Services, Food and Drug Administration, Center for Drug Evaluation and Research, Center for Biologics Evaluation and Research, and Center for Devices and Radiological Health (2022) Patient-focused drug development: Methods to identify what is important to patients; Guidance for industry, food and drug administration staff, and other stakeholders. https://www.fda.gov/media/131230/download. Accessed 25 Oct 2023

[6] Wild D, Grove A, Martin M, Eremenco S, McElroy S, Verjee-Lorenz A, Erikson P (2005) Principles of good practice for the translation and cultural adaptation process for Patient-Reported outcomes (PRO) measures: report of the ISPOR task force for translation and cultural adaptation. Value Health 8:94–104. 10.1111/j.1524-4733.2005.04054.x 15804318 10.1111/j.1524-4733.2005.04054.x

[7] Eremenco S, Pease S, Mann S, Berry P (2018) PRO consortium’s process subcommittee. Patient-Reported outcome (PRO) consortium translation process: consensus development of updated best practices. J Patient Rep Outcomes 2:12. 10.1186/s41687-018-0037-6 10.1186/s41687-018-0037-6 PMC5934912 29757299

[8] Mowlem F, Elash C, Dumais K, Haenel E, O’Donohoe P, Olt J, Kalpadakis-Smith A, James B, Balestrieri G, Becker K, Newara M, Kern S, Electronic Clinical Outcome Assessment Consortium (2024) Best practices for the electronic implementation and migration of Patient-Reported outcome measures. Value Health 27:79–94 7 37879401 10.1016/j.jval.2023.10.007

[9] Acquadro C, Patrick DL, Eremenco S, Martin M, Kulis D, Correia H, Conway K (2018) Emerging good practices for translatability assessment (TA) of Patient-Reported outcome (PRO) measures. J Patient Rep Outcomes 2:8 10.1186/s41687-018-0035-8 PMC5935017 29757337

[10] Coons SJ, Gwaltney CJ, Hays RD, Lundy JJ, Sloan JA, Revicki DA, Lenderking WR, Cella D, Basch E (2009) Recommendations on evidence needed to support measurement equivalence between electronic and paper-based patient-reported outcome (PRO) measures: ISPOR ePRO good research practices task force report. Value Health 12:419–429 19900250 10.1111/j.1524-4733.2008.00470.x

[11] McKown S, Acquadro C, Anfray C, Arnold B, Eremenco S, Giroudet C, Martin M, Weiss D (2020) Good practices for the translation, cultural adaptation, and linguistic validation of clinician-reported outcome, observer-reported outcome, and performance outcome measures. J Patient Rep Outcomes 4:489. 10.1186/s41687-020-00248-z 10.1186/s41687-020-00248-z PMC7642163 33146755

[12] Clancy C, Crane A, Millman J (2018) eCOA Translations and Cultural Adaptation. Best Practices and Efficiencies. https://www.yprime.com/ecoa-translations-cultural-adaptation/. Accessed 28 Feb 2022

[13] Oxford University Innovation (Nov 2016) Patient Reported Outcomes: From Paper to ePROs. Good Practice Guide for Migration. https://innovation.ox.ac.uk/wp-content/uploads/2016/05/ePRO_guide_2016.pdf. Accessed 20 Jan 2022

[14] Romero H, DeBonis D, O'Donohoe P et al (2022) Electronic Patient-Reported Outcome Consortium and the Patient-Reported Outcome Consortium. Recommendations for the Electronic Migration and Implementation of Clinician-Reported Outcome Assessments in Clinical Trials. Value Health 25: 1090-1098. 10.1016/j.jval.2022.02.012 10.1016/j.jval.2022.02.012 35379564

[15] Sweeney E, Kelley T (2014) The importance of migration assessments: eCOA translations and linguistic validation. Value Health 17:A574. 10.1016/j.jval.2014.08.1927 27201925 10.1016/j.jval.2014.08.1927

[16] Regulation EU (2016) 2016/679 of the European Parliament and of the Council of 27 April 2016 on the protection of natural persons with regard to the processing of personal data and on the free movement of such data, and repealing. Directive 95/46/EC (General Data Protection Regulation) https://eur-lex.europa.eu/eli/reg/2016/679/oj. Accessed 23 Nov 2023

[17] Gordon S, Crager J, Howry C, Barsdorf AI, Cohen J, Crescioni M, Dahya B, Delon P, Knaus C, Reasner DS, Vallow S, Zarzar K, Eremenco S (2022) Electronic Patient-Reported (ePRO) consortium, PRO consortium. Best practice recommendations: user acceptance testing for systems designed to collect clinical outcome assessment data electronically. Ther Innov Regul Sci 56:442–453. 10.1007/s43441-021-00363-z 35233726 10.1007/s43441-021-00363-z PMC8964567

[18] Snowdon DA, Srikanth V, Beare R, Noeske KE, Le E, O’Bree B, Andrew N (2023) Acceptability of the routine use and collection of a generic patient reported outcome measure from the perspective of healthcare staff: a qualitative study. J Patient Rep Outcomes 7:623–633. 10.1186/s41687-023-00617-4 10.1186/s41687-023-00617-4 PMC10390450 37522943

