# Supplementary material for: Principles of good practice for translation of electronic clinical outcome assessments
Source: J Patient Rep Outcomes. 2025 Mar 4;9:26. doi: 10.1186/s41687-025-00859-4 (PMC11880485; doi:10.1186/s41687-025-00859-4)
Supplement: Supplementary file 1 — Supplementary Material 1 [file 41687_2025_859_MOESM1_ESM.docx]

# **APPENDIX A: LSP and eCOA Provider Survey**

# **ISOQOL TCA-SIG SURVEY (2019):**

**Identifying Views and Processes Related to Electronic Clinical Outcome Assessment (eCOA) Methodology when Combined with Translations**

**What is this survey about?**

The International Society for Quality of Life Research Translation and Cultural Adaptation Special Interest Group (ISOQOL TCA-SIG) is developing a “best practices” guidance paper on translations related to electronic clinical outcome assessment (eCOA) measures such as electronic patient reported outcome (ePROs), observer-reported outcome (eObsROs), clinician-reported outcome (eClinROs), and performance outcome measures (ePerfOs). This research aims to create and outline a step-by-step descriptive approach of the methods to be used to translate non-instrument text needed for eCOA implementation. We realize there is a gap in the literature and are aiming to provide more specific guidance on this topic, with your help.

This survey contains questions about eCOA and translation project setup, the translation process, developer involvement, and any future wishlist for this type of research. You will also be asked to provide some basic information about your organization, including name, location, and number of projects.

**How long will this survey take to complete?**

It will take you approximately 10 minutes to complete this survey.

**Who is participating?**

eCOA and translation providers who work on eCOA research are participating. We ask for one response per organization. ***Note that all participating organizations will be acknowledged in the final guideline dissemination manuscript publication to thank them for their collaboration.*** We value your experience and greatly appreciate you taking the time to participate in this survey!

If interested in participating and ready to complete the survey, please click on **yes** to get started.

- Yes, interested [allows to proceed to survey]
- No, I do not wish to continue

***[new page]***

**PROVIDER DEMOGRAPHICS**

***[each question below on a new page]***

| 1. Would you like to participate in this survey? | 1. Yes 2. No   *End survey if No* |
| --- | --- |
| 2. Are you a ….? | 1. Translation provider? 2. eCOA provider?   *Each will get a separate survey from here* |
| 3. Please provide your company/organization name: | *Free text* |
| 4. Please provide main office Location/s (city, country): | *Free text*  *Show #3 and 4 on same page.* |
| 5. Approximately how much of your overall translation project work is US based versus global (other countries)? | 1. Mostly non-US (international) 2. About half non-US and half US 3. Mostly US only |
| 6. Approximately much of your eCOA translation work is funded by a(n) …...? (Total = 100%) | 1. Pharmaceutical company % 2. Private healthcare company % 3. CRO % 4. eCOA vendor % 5. Translation vendor % 6. Academia % 7. Other % |
| 7. How experienced is your organization translating eCOAs? | 1. Very experienced 2. Experienced 3. Some experience 4. No experience   *One answer only allowed* |
| 8. How experienced is your organization migrating COAs from paper to electronic formats or creating de novo eCOA instruments? | 1. Very experienced 2. Experienced 3. Some experience 4. No experience |

**PROJECT SET-UP**

1. When you have a project that includes eCOA migration and translation work simultaneously for the same instrument, who is involved throughout the duration of the project lifecycle (start to finish) from your team? Please select all who are involved in the process.
   1. Project manager/coordinator
   2. Linguist
   3. Scientific expert
   4. Cultural expert
   5. Clinical expert
   6. eCOA expert
   7. Research scientist
   8. Other role(s) not listed
   9. Not applicable
2. When migrating (paper to eCOA) and translating the same instrument at the same time, do you have any standard quality control checks and/or processes in place throughout the project lifecycle?
3. YES, quality check of eCOA migration (show 11a if YES)
4. YES, quality check of translations (show 11a if YES)
5. YES, other quality check (show 11a if YES)
6. No specific quality control checks
7. Not applicable

10a. You mentioned “YES” to standard quality checks and/or processes in the earlier question. Please provide more detail: ______________________ [FREE TEXT]

1. When using existing translations to prepare to migrate a COA to an electronic platform, do you evaluate the measure (e.g., feasibility of wording changes to electronic, or translatability assessment, etc.) before you migrate and translate?
   1. Yes, we conduct a translatability assessment
   2. Yes, we conduct a feasibility assessment to change to electronic (e.g., select vs circle)
   3. Yes, we do both a translatability and feasibility assessment
   4. Other (show 12a if YES)
   5. No evaluation before migration
   6. Not applicable

11a. You mentioned “Yes, other” in the earlier question related to evaluating the measures before migrating and translating. Please provide more detail: ___________________ [FREE TEXT]

1. When working on a standardized COA from a developer to migrate the instrument (paper to electronic), how often do you get specific instructions from the vendor about how to do that?
2. Always
3. Often
4. Sometimes
5. Rarely
6. Never
7. Not applicable

12a. How helpful would it be to get specific instructions on electronic migration from the developer?

1. Very helpful
2. Helpful
3. Somewhat helpful
4. Not very helpful
5. Not helpful at all
6. When working with a developer to translate and migrate to an electronic platform a standardized COA (licensed from developer), how often do you receive the COA in Word format (as opposed to a pdf, for example)?
   1. Always
   2. Often
   3. Sometimes
   4. Rarely
   5. Never
   6. Not applicable
7. How helpful would it be to get the COA instrument from the developer in Word format when translating and migrating to electronic format?
8. Very helpful
9. Helpful
10. Somewhat helpful
11. Not helpful
12. Not applicable
13. If a standardized template (e.g., an Excel sheet) that streamlines the eCOA and translation process existed (e.g. flowchart, excel sheet explaining steps, etc.), do you think that would be helpful to have?
    1. Very helpful
    2. Helpful
    3. Somewhat helpful
    4. Not very helpful
    5. Not helpful at all
14. When implementing multiple COAs on a single electronic platform (e.g., tablet or BYOD which could have been obtained from more than one developer), do you find that it is important to create visual harmony /consistency between the instruments for better patient engagement?
    1. Yes, visual harmony/consistency is important
    2. No, visual harmony/consistency isn’t that important
    3. Not sure

**TRANSLATION PROCESS**

17. When working on a translation and conducting electronic migration of the same COA, please select all the steps you undertake below in your department/company.

- 1. Feasibility assessment to migrate to electronic
  2. Single forward translation
  3. Dual forward translation
  4. Reconciliation of forward translations
  5. Single backward translation
  6. Dual backward translation
  7. Reconciliation of backward translations
  8. Back translation review
  9. Harmonization
  10. Clinical review
  11. In-country review
  12. Developer review
  13. Cognitive interviews with target population
  14. Other steps not listed
  15. Not applicable

*More than one answer allowed*

18. What process do you undertake to translate navigational text that are necessary for eCOA implementation, but which are not part of the instrument? (For example, adding the *Back* and *Next* button text on an electronic device). Please select all steps that apply

- 1. Translatability assessment
  2. Feasibility assessment to migrate to electronic
  3. Single forward translation
  4. Dual forward translation
  5. Reconciliation of forward translations
  6. Single backward translation
  7. Dual backward translation
  8. Reconciliation of backward translations
  9. Back translation review
  10. Harmonization
  11. Clinical review
  12. In-country review
  13. Developer review
  14. Cognitive interviews with target population
  15. Other steps not listed
  16. Not applicable

19. Based on your experience, is there a difference in how long it takes to complete COA translations (including forward and back translations), from start to finish, when an instrument hasn’t been migrated before, compared to paper COA translation?

1. Yes, electronic COA migration and translation takes longer (show 21a if YES)
2. Yes, paper translation takes (show 21a if YES)
3. There is no difference
4. Not sure

19a. You answered “Yes” to the earlier question about whether there is a difference in how long it takes to complete electronic vs paper translation. Please explain your response by adding an approximate amount of time longer (e.g. number of days/weeks): ________________________________________ *[FREE TEXT]*

- 1. What top three factors most impact timing when developing eCOA translations compared to paper COA translations? The provider
  2. Approval time from clients/other stakeholders
  3. Time of the year
  4. Programming of the COA
  5. Translation process
  6. Length of instrument
  7. Quality control checks (translation and/or migration)
  8. Initial feasibility (translatability assessment or migration wording changes)
  9. Other

*Allow to choose top 3 only/ randomize except for Other*

1. Which research methods do you generally undertake when conducting linguistic validation of the eCOA translations with participants?
2. Cognitive interviews (show 23b if a chosen)
3. Concept elicitation
4. In-person interviews
5. Telephone interviews
6. Focus groups
7. Usability testing (e.g., tablet, handheld, web) to present instrument content
8. Use of Paper copy screenshots to present instrument content
9. Other, please specify *More than one answer allowed- on same screen show below 23a*

If chose (a) above, ask:

20b. What is the typical sample size that you use to conduct ***cognitive interviews*** for linguistic validation of an electronic COA for the population of interest? ________________________________ *[FREE TEXT]*

1. Do you normally retest an electronic COA with your participants after changes are implemented from patient feedback (e.g. conduct research in waves) before finalization?
   1. Yes (show 21a if Yes)
   2. No
   3. Not applicable

21a. If yes, are the same participants used for retesting, or are new ones recruited?

- - 1. Same participants
    2. New participants
    3. Combination of new and existing participants
    4. Not sure

1. When conducting research on the migration of a COA and translating the same COA, how do you select your population?
   1. Match as closely as possible to specific trial population of interest
   2. General population
   3. Other, please specify
   4. Not applicable

**DEVELOPER INVOLVEMENT**

1. When using a standardized instrument, how often do you involve instrument developers in the process of translation of their instrument?
   - Always
   - Often
   - Sometimes
   - Rarely
   - Never
   - Not applicable
2. When using a standardized instrument, how often do you involve instrument developers in the process of migration from paper to electronic of their instrument?
   1. Always
   2. Often
   3. Sometimes
   4. Rarely
   - Never
   - Not applicable
3. Based on your experience, how often does the instrument developer get involved in the review of the instrument during and after electronic migration (e.g. by giving input about the format and content where the copyright information should be placed, how many questions per page, if the instructions should be present on every page or not, if text should be presented in a table/free floating, having leader dots, etc.)?
   1. Always
   2. Often
   3. Sometimes
   4. Rarely
   5. Never
   6. Not applicable
4. Based on your experience, how many rounds of review by an instrument developer are usually needed to finalize an instrument? *[number, 0-10]*
5. Based on your experience, how many rounds of review by an eCOA provider are usually needed to finalize an instrument? *[number, 0-10]*

**FUTURE STATE**

1. What do you wish existed that does not yet exist in the process as an industry standard when working to migrate and translate an instrument at the same time?
   1. Editable file (e.g., Word, Rich Text Format)
   2. Developer instructions
   3. List of concepts
   4. Other (if selected, show 31a)

31a. You answered “Other” what you wish existed as an industry standard. Please specify the “Other” category: __________________________ *[FREE TEXT]*

Thank you, almost done!

If you are interested in keeping up to date with the dissemination of this research, please provide your contact details here: *FREE TEXT*

*First name: ____________*

*Last name: ___________*

*Email: ____________*

[NEW PAGE]

Thank you for your participation!

This survey is now closed

# **APPENDIX B: Developer/ copyright Holder Survey**

**ISOQOL TCA-SIG SURVEY (2021):**

**Identifying Views and Processes Related to Electronic Clinical Outcome Assessment (eCOA) Methodology when Combined with Translations**

(**Instrument Developer Experience)**

**What is this survey about?**

The International Society for Quality of Life Research Translation and Cultural Adaptation Special Interest Group (ISOQOL TCA-SIG) is developing a best practice~~s~~ recommendations paper on streamlining the process of translation and migration of electronic clinical outcome assessments (eCOA) from a paper to an electronic format. We realize there is a gap in the literature and are aiming to provide more specific recommendations on this topic, with your help.

This survey seeks to gain the knowledge and understanding of your experience of the migration and translation of clinical outcome assessments (instruments) from paper to electronic (i.e. handheld, tablet, bring your own device [BYOD] or web).

**Collaboration:**

The C-Path eCOA: Getting Better Together Initiative (GBTI) on Flexible Implementation is collaborating with the TCA-SIG on this effort and have added three questions in the last section of the survey. They are working on a manuscript to address streamlining and simplifying eCOA implementation in a broader context.

**How long will this survey take to complete?**

It will take you approximately 20 minutes to complete this survey.

**Who is participating?**

This survey is targeted towards copyright holders, instrument developers~~,~~ scientific experts, and organizations who deal with licensing and reviews of instruments. We ask for one response per license holder or organization.

**Why take part?**

All participating organizations or developers will be acknowledged by name (except if otherwise notified) in the final best practices publication to thank them for their input. We value your experience and greatly appreciate you taking the time to participate in this survey!

Are you still interested in participating in the survey?

1. Yes, I am interested. If yes, do you accept your name be acknowledged in the final best practice publication? An answer of ‘no’ does not preclude you from responding to this survey.
   1. Yes
   2. No

1. No, I am not interested. If No, “Thank you for participating. The survey is now closed.”~~.~~

**SECTION I: OVERVIEW**

| **#** | **Question** | **Details** |
| --- | --- | --- |
| 1. | Are you responding as a ….? Please choose one option that best describes your role | 1. Copyright holder / instrument developer/instrument licensor or distributor 2. Scientific expert involved in the process of migrating and translating standardized licensed instruments 3. Other   If Other, please provide a box for them to specify their role. |
| 2. | Approximately how many instruments do you or your organisation licence or hold the copyright for? | 1. 1-3 2. 4-7 3. 8-10 4. 11+ |
| 3. | On average, how long does it take, from start to finish to license one instrument with your organization? Think about the time from when a request is made to the time an instrument is delivered to the requestor, including the time for the contract to be executed and the instrument to be delivered, where applicable. | - 0-1 week - 1-2 weeks - 2-4 weeks - 4-6 weeks - 6-8 weeks - 8-12 weeks - 13 weeks or more - I am not sure |

**SECTION II: IMPLEMENTATION QUESTIONS & DETAILS**

| **4.** | **Have you (or a member of your team/organization) been involved at any stage in the electronic migration (transfer from a paper to an electronic format) of your instrument(s)?**   1. Yes 2. No [go to question 7]   4a. If yes: **Please select all type(s) of projects you have been involved in for the electronic migration of your instrument:** May select more than one response.   1. Source language 2. Existing translations/adaptations (e.g., French for Switzerland) 3. New translations directly followed by migration 4. None of the above |
| --- | --- |
| 5. | **Please select the stage(s) in which you have been involved in electronic migration from the list below.** |

| □ | Initial discussion / preparation phase before migration process begins, including the preparation of the text specific for electronic migration, which needs to be translated~~.~~ |
| --- | --- |
| □ | Migratability assessment- necessary changes for electronic migration to content (e.g., changes in wording of instructions, navigational text)~~.~~ |
| □ | Migratability assessment - necessary changes for electronic migration to layout (e.g., one item/multiple items per screen, layout of response options, length of translations)~~.~~ |
| □ | Proofreading migrated translations |
| □ | Screenshot review |
| □ | Review of cognitive interview results |
| □ | Review of usability testing / expert review results |
| □ | Final review after implementing all changes |
| □ | Other: Please specify |

| **6.** | **How often are you (or a member of your team/organization) involved in the electronic migration process of your instrument(s)?** |
| --- | --- |

| □ | □ | □ | □ | □ |
| --- | --- | --- | --- | --- |
| All the time | Most of the time | Sometimes | Rarely | Not at all |

| **7.** | **In your opinion, how important is it for you to be involved in the electronic migration process as a license/copyright holder/scientific expert?** | | | | |
| --- | --- | --- | --- | --- | --- |
| □ | | □ | □ | □ | □ |
| Very  important | | Moderately important | Somewhat important | Slightly  important | Not at all important |

| **8.** | **Do you normally have a set of requirements or recommendations that you provide to licensees for instrument migration from paper to electronic?** |
| --- | --- |
|  | Yes □ |
|  | No □ [go to question 12.] |
| **9.** | **Do you have requirements or recommendations for device type or screen size (e.g., tablet, smartphone or web)?** |
|  | Yes □ |
|  | No □ [go to question 12.] |
| **10.** | **Do your requirements or recommendations include instructions on how to adjust wording of your instruments for electronic implementation?** |
|  | Yes □ |
|  | No □ [go to question 12] |

| **11.** | **Who do you provide this set of requirements or recommendations to? (select all that apply)** |
| --- | --- |
|  | 1. Requestor (e.g., sponsor, CRO, researcher) |
|  | 1. Licensee 2. eCOA provider 3. Translation provider |

| **12.** | **How often do you receive instrument screenshots for review from a licensee?** |
| --- | --- |

| □ | □ | □ | □ | □ | □ |
| --- | --- | --- | --- | --- | --- |
| All the time | Most of the time | Sometimes | Rarely | Not at all | Do not know |

| **13.** | **How often do you observe deviations from your electronic migration requirements or recommendations and the screenshots received (e.g., from eCOA providers), if applicable to you?** |
| --- | --- |

| □ | □ | □ | □ | □ | □ |
| --- | --- | --- | --- | --- | --- |
| All the time | Most of the time | Sometimes | Rarely | Not at all | Not applicable |

| **14.** | **How often do eCOA platform limitations hinder the eCOA provider’s ability to follow your requirements or recommendations, if applicable to you?** |
| --- | --- |

| □ | □ | □ | □ | □ | □ |
| --- | --- | --- | --- | --- | --- |
| All the time | Most of the time | Sometimes | Rarely | Not at all | Not applicable |

| **15.** | **In case any deviations are expected to have a substantial impact on the equivalence in interpretation of the instrument between paper and electronic, do you….? (Only ask if ‘All the time’, ‘Most of the time’, ‘Sometimes’ and ‘Rarely’ to Q.14.)** |
| --- | --- |

|  | 1. Provide alternatives that are acceptable to you as an instrument developer 2. Refuse instrument migration if acceptable alternatives are not / cannot be implemented 3. Other (please describe) |
| --- | --- |

| **16.** | **In your experience, which aspects of the electronic migration of an instrument have been problematic? Please select any that apply.** | |
| --- | --- | --- |
| 1. Initial assessment of changes needed – adaptations and additions related to delivery via electronic platform. | |  |
| □ | Migratability assessment (considering any changes for electronic migration) |  |
| **□** | Approved modifications in wording of instrument instructions (e.g., summarizing in order to fit the device screen) |  |
| □ | Approved modifications in item wording and response options wording |  |
| □ | Unapproved modifications to items or instructions |  |
| □ | Presenting instructions / recall period |  |
| □ | Presentation of copyright information |  |
| 1. Formatting the questionnaire | |  |
| □ | Number of items per screen |  |
| □ | Formatting text (e.g., underline, **bold**, *italics*, coloured text, CAPITALS) |  |
| □ | Layout of response options (e.g., vertically / horizontally/ spacing in between them) |  |
| □ | Scrolling, zooming in and out (seeing all response options on one screen) |  |
| □ | Screen size (original vs. translations) |  |
| 1. Flow of the questionnaire | |  |
| □ | Skipping items, if allowed |  |
| □ | Skip patterns / branching logic |  |
| □ | Final overview page showing all items and selected responses |  |
| □ | Answering missed items (e.g., navigational buttons, pop-up indicating a response was not entered) |  |
| □ | Progress bar showing where the respondent is in the eCOA |  |

| **17.** | **Which particular aspect(s) of the combined process of translation and electronic migration do you think would be most important to be involved in? Please select any that apply:** |
| --- | --- |
| □ | Initial discussion / preparation phase before migration process begins, including the preparation of the text specific for electronic migration, which needs to be translated |
| □ | Translatability assessment - necessary changes for linguistic implementation of content (e.g., changes in wording of instructions, navigational text, their ease of translation) |
| □ | Migratability assessment - necessary changes for electronic migration of layout (e.g., one item/multiple items per screen, layout of response options) |
| □ | Proofreading migrated translations |
| □ | Screenshot review |
| □ | Review of cognitive interview results |
| □ | Review of usability testing~~.~~ |
| □ | Review of expert review results |
| □ | Final review after implementing all changes |
| □ | Other, please specify |

| **18.** | **Would you find a set of consensus best practice requirements or recommendations useful when migrating and translating a licensed instrument?** |
| --- | --- |
|  | Yes □ |
|  | No □ |

| **19.** | **Do you have any other comments/suggestions related to streamlining the electronic migration and translation processes that you would like to share? (Please respond in English for the ease of the writing team).** [Free Text – English only] |
| --- | --- |
|  | |

[NEW PAGE]

Thank you for your participation! The results of this survey will be disseminated in a publication via ISOQOL.

This survey is now closed.
